# Assessment of Nociception and Inflammatory/Tissue Damage Biomarkers in a Post-COVID-19 Animal Model

**DOI:** 10.3390/ijms27010359

**Published:** 2025-12-29

**Authors:** Eva M. Sánchez-Robles, Carmen Rodríguez-Rivera, Nancy Paniagua Lora, Esperanza Herradón Pliego, Carlos Goicoechea Garcia, Lars Arendt-Nielsen, Cesar Fernández-de-las-Peñas, Visitación López-Miranda

**Affiliations:** 1 Department of Basic Health Sciences, Universidad Rey Juan Carlos, 28922 Madrid, Spain; eva.sanchez@urjc.es (E.M.S.-R.); carmen.rodriguez@urjc.es (C.R.-R.); nancy.paniagua@urjc.es (N.P.L.); esperanza.herradon@urjc.es (E.H.P.); carlos.goicoechea@urjc.es (C.G.G.); visitacion.lopezmiranda@urjc.es (V.L.-M.); 2 Unidad Asociada al Instituto de Química Medica (IQM) del Consejo Superior de Investigaciones Científicas (CSIC), Universidad Rey Juan Carlos, 28922 Madrid, Spain; 3 High Performance Research Group in Experimental Pharmacology (Pharmakom-URJC), Universidad Rey Juan Carlos, 28922 Madrid, Spain; 4 Center for Neuroplasticity and Pain (CNAP), Sensory-Motor Interaction (SMI) Center, Department of Health Science and Technology, Faculty of Medicine, Aalborg University, DK-9220 Aalborg, Denmark; lan@hst.aau.dk; 5 Department of Gastroenterology & Hepatology, Mech-Sense, Clinical Institute, Aalborg University Hospital, DK-9000 Aalborg, Denmark; 6 Steno Diabetes Center North Denmark, Clinical Institute, Aalborg University Hospital, DK-9000 Aalborg, Denmark; 7 Department of Physical Therapy, Occupational Therapy, Physical Medicine and Rehabilitation, Universidad Rey Juan Carlos, 28922 Madrid, Spain

**Keywords:** Post-COVID-19, nociception, pain, inflammation biomarkers, female mice, hACE2

## Abstract

Five years after the onset of the SARS-CoV-2 pandemic, post-COVID-19 condition continues to affect millions of subjects with persistent symptoms that significantly impair quality of life. Post-COVID-19 pain, particularly in women, has emerged as a frequent yet underestimated symptom. The validation and identification of animal models that reproduce persistent symptoms after an acute SARS-CoV-2 infection is crucial for a better understanding of the underlying mechanisms. The aim of the current study was to evaluate thermal nociception, biomarkers of inflammation, and nerve tissue damage in a female animal model of post-COVID-19 condition. A SARS-CoV-2 infection model was established by intranasal administration of the Omicron variant (BA.1.17 lineage) in transgenic female C57BL/6 mice expressing the human ACE2 receptor (hACE2). Nociception was assessed using the hot-plate test for 28 days post-infection. Afterwards, animals were sacrificed to analyze plasma inflammatory biomarkers by multiplex analysis. In addition, IL-6, IL-18, and IL-1β expression were evaluated by immunohistochemistry to analyze neural inflammation in the saphenous nerve. The results revealed that heat nociceptive thresholds in infected mice did not significantly differ from those of non-infected, but a trend toward lower thresholds was observed in the infected group (days 14 and 28 post-infection). In addition, a slight modification in pro- and anti-inflammatory cytokines/chemokines in plasma was detected, but no changes in the expression of IL-6, IL-1β, or IL-18 were observed in the saphenous nerve. Based on all the analyses conducted, infection with the Omicron variant of SARS-CoV-2 did not induce thermal sensitization in animals nor alterations in the expression of inflammatory biomarkers in the saphenous nerve. Finally, a slight state of systemic inflammation was present in the infected animals. The absence of detectable changes in this animal model underscores the need for further research to clarify the discrepancies observed in human patients and to explore alternative pathways potentially involved in post-COVID-19 pain syndromes.

## 1. Introduction

As of December 2025, five years after the appearance of the Severe Acute Respiratory Syndrome Coronavirus 2019 2 (SARS-CoV-2), with close to 779 million infections and over 7.2 million deaths reported, the coronavirus disease 2019 (COVID-19) represents a global health crisis with devastating impact on healthcare systems [1]. Evidence suggests that up to 25–30% of individuals who had survived an acute SARS-CoV-2 infection exhibit long-lasting symptoms two or three years after the infection [2,3]. In addition, the presence of post-COVID-19 symptoms significantly affects individuals’ health-related quality of life [4]. Wang et al. observed that from those individuals experiencing post-COVID-19 symptoms during the first year after the acute infection, only 5% received an appropriate diagnosis of long COVID [5].

The lack of a proper diagnosis can be related to the fact that manifestations of post-COVID-19 condition are heterogeneous, since individuals can be grouped in different clusters according to their predominant symptoms [6]. Thus, a “musculoskeletal pain cluster” has been identified [6], and the term “post-COVID pain syndromes” has also been proposed [7]. The prevalence of post-COVID-19 pain is highly heterogeneous in different studies. Fernández-de-las-Peñas et al. found that up to 20% of COVID-19 survivors reported post-COVID-19 pain symptomatology in different areas of the body and at different follow-ups during the first months after the infection [8]. This prevalence rate was confirmed in a later meta-analysis [9]. Nevertheless, it should be noted that the studies specifically investigating the prevalence of post-COVID-19 pain reported higher prevalence rates (40% to 60%) than the above-mentioned meta-analysis, which included epidemiological studies on general post-COVID-19 symptomatology [10,11,12,13].

Thus, a proper understanding of the operating mechanisms behind each post-COVID-19 symptom is crucial for a personalized medicine approach. In this context, animal models have emerged as potential tools to investigate the pathogenesis of COVID-19 and post-COVID-19 condition. Different animal models, including hamsters, non-human primates, mice, rats, ferrets, rabbits, and cats, have been proposed to simulate SARS-CoV-2 infection [14]. Different animal models, e.g., non-human primates, golden Syrian hamsters, or transgenic/virus-adapted mice, have proven to be useful for studying viral persistence, immune dysfunction, intestinal dysbiosis, or neurovascular changes typical of COVID-19 multiorgan manifestations [15]. It has been documented that the best animal model mimicking an acute SARS-CoV-2 infection seems to be the use of multigene humanized mice C57BL/6J [16]. An animal model infecting hACE2 female mice C57BL/6J with the Omicron (lineage BA.1.17) SARS-CoV-2 variant has been proposed to mimic findings in humans, since it was able to detect viral RNA and significantly elevated levels of pro-inflammatory interleukins in the lungs at the acute phase (4 days after) but not in the post-COVID-19 phase (15 days post-infection) [17]. Thus, the presence of detectable viral SARS-CoV-2 RNA at the acute COVID-19 phase but not after mimics current findings on humans, where no detectable viral RNA has been identified in individuals with post-COVID-19 condition [18,19]. Accordingly, studies using this animal model infected with the current variant of concern, i.e., Omicron, are required to better understand the development of post-COVID-19 symptomatology.

Since female sex is a factor associated with a higher risk of developing post-COVID-19 pain, as well as other post-COVID-19 symptoms [20], the control of biological sex in animal models is crucial. Thus, most studies developing animal models have used male animals [21]. Accordingly, this study used a model of SARS-CoV-2 infection with female transgenic mice expressing the human angiotensin-converting enzyme 2 (ACE2) receptor. The aims of the current study were to assess nociception to thermal noxious stimuli to identify possible alterations in nociceptive thresholds following Omicron variant SARS-CoV-2 infection and to analyze different biomarkers of inflammation and tissue damage to contribute to a better understanding of the pathophysiological mechanisms underlying post-COVID-19 pain.

## 2. Results

### 2.1. Survival Rate and Body Weight

**Figure 1:**
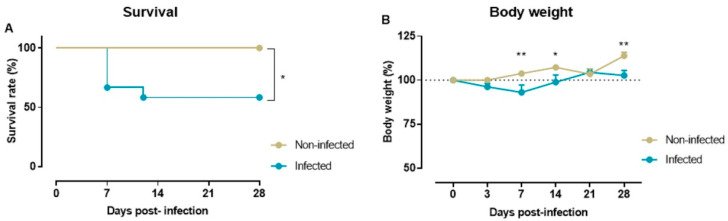
Survival curves (**A**) and body weight (**B**) of infected (n = 7) and non-infected (n = 11) animal groups. The body weight of mice at days 3, 7, 14, 21, and 28 post-infection is expressed as a percentage of their initial body weight on day 0. Data are presented as mean ± SEM * *p* < 0.05; ** *p* < 0.01.

Of the twelve mice infected with SARS-CoV-2, five had to be euthanized on the first week post-infection according to humane endpoints for animal experimentation (four mice on day 4 and one on day 8 post-infection) after exhibiting weight loss (average loss of approximately 10%). These animals were excluded from nociception and biomarker analyses because they were removed from the study. Survival analysis showed a rate of 58.3% in the infected group (7/12 mice), whereas all animals in the non-infected control group (0/11 mice) survived (Figure 1A). Differences in survival rate were statistically significant (*p* = 0.0178, log-rank Mantel–Cox test).

The body weight of each animal was monitored at 3, 7-, 12-, 21-, and 28-days post-infection. Two-way repeated measures ANOVA found a significant group*time interaction (F(5,80) = 3.675, *p* = 0.005). Sidak’s multiple comparisons test revealed significant between-groups differences at 7-, 14-, and 21-days post-infection (*p* = 0.0034, *p* = 0.0385, *p* = 0.003, respectively): infection with SARS-CoV-2 induced a significant loss of body weight at 7 days post-infection that was not recovered up to 14 days post-infection (Figure 1B).

### 2.2. Nociception to Heat Stimulus

**Figure 2:**
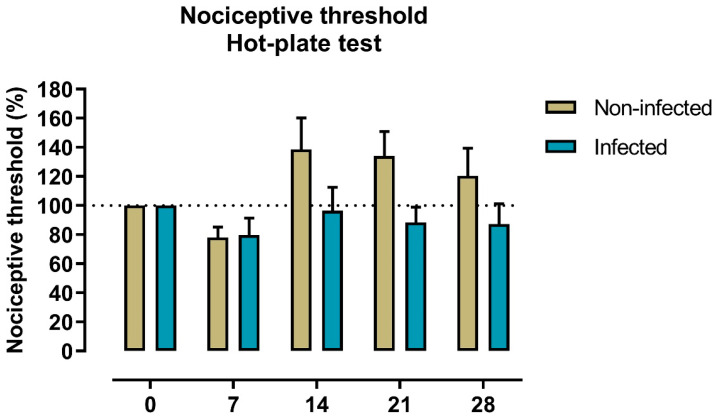
Nociceptive thresholds to heat stimulus in non-infected (n = 11) and infected (n = 7) female mice. Bars represent the nociceptive threshold (mean ± SEM) corresponding to the latency of the first observable response on the hot plate at 55 °C. Latencies were normalized to baseline values (Day 0 considered 100%).

The potential development of pain over time post-infection was assessed by measuring nociceptive thresholds to heat on the hot-plate test once a week until day 28 post-infection. The latency (s) before infection (day 0) for non-infected and infected animals were 7 and 8.4 s, respectively. These values correspond to a nociceptive threshold of 100%. When nociceptive thresholds were compared at 7-, 14-, 21-, and 28-days post-infection, a two-way repeated measures ANOVA found no significant group*time interaction (F(4,64) = 1.536, *p* = 0.202). Although there was a tendency for the infected group to exhibit lower nociceptive thresholds from day 14 to 28 post-infection, Sidak’s multiple comparisons test showed no statistical differences (Figure 2).

### 2.3. Plasma Levels of Cytokines

No significant differences between cytokine/chemokine levels in plasma samples of SARS-CoV-2-infected animals and non-infected control mice were observed (all, *p* > 0.05, Table 1).

**Figure 3:**
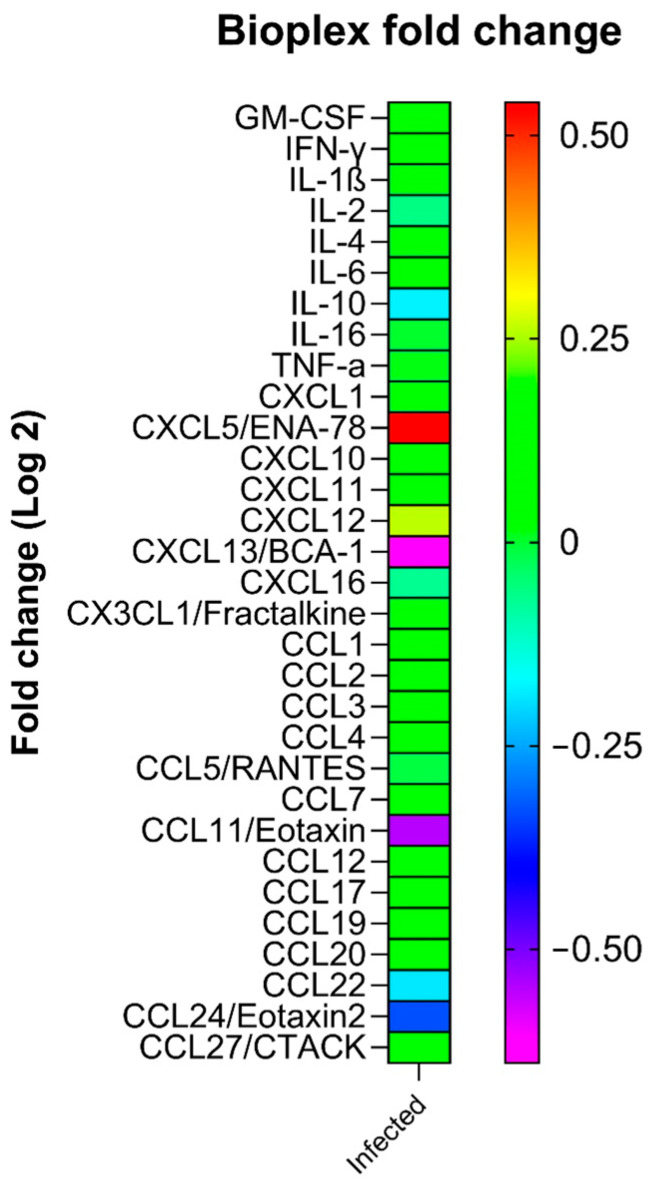
Heat fold map of trends in expression of analyzed cytokines/chemokines in infected and non-infected animals. Fold changes represent the log2 transformation of the ratio between the concentration of each sample of the infected group and the mean concentration of the non-infected group.

To further investigate cytokine and chemokine expression, trends were assessed using a fold-change heat map (Figure 3). This analysis indicated a potential increase in CXCL5/ENA-78 expression and, to a lesser extent, CXCL12 in plasma samples from infected animals compared to non-infected controls. Conversely, a trend toward reduced expression of CCL11/Eotaxin, CCL24/Eotaxin-2, and CXCL13/BCA-1, as well as a slight decrease in IL-10 and CCL22, was observed in infected animals compared to non-infected controls. No consistent variation was detected in the expression of the remaining cytokines and chemokines between the two groups.

### 2.4. Expression of IL-6, IL-1β, and IL-18 in the Peripheral Nerve

**Figure 4:**
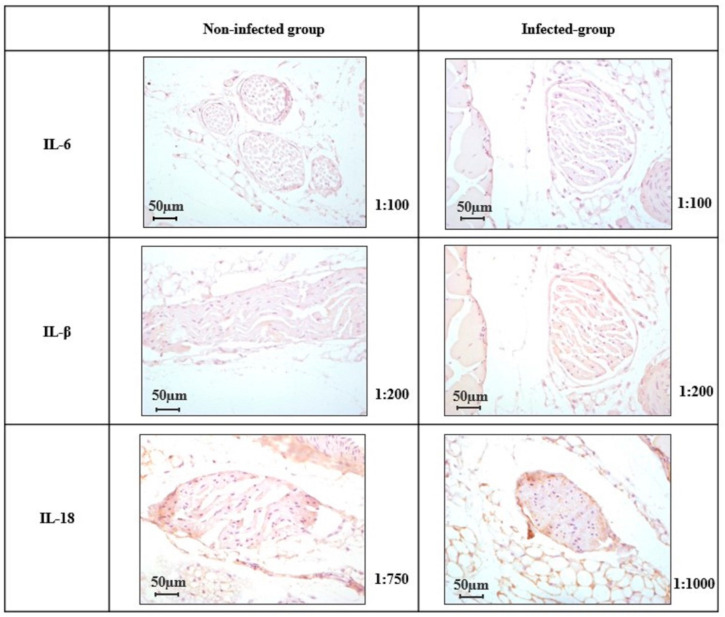
Protein expression of IL-6, IL-1β, and IL-18 in the saphenous nerve in non-infected and infected female mice. Representative images of immunohistochemistry (20×) of IL-6 expression, IL-1β expression, and IL-18 expression in the saphenous nerve from infected and non-infected female mice.

In the saphenous nerve preparations, no expression of IL-6, IL-1β, and IL-18 was detected in either infected or non-infected groups of animals (Figure 4).

## 3. Discussion

This preclinical post-COVID-19 study using female transgenic mice infected with the SARS-CoV-2 Omicron variant (BA.1.17 lineage) revealed no significant alterations in nociception to heat nociceptive stimulus or in the expression of related biomarkers in saphenous nerve tissue 28 days post-infection. However, an inflammatory environment in animals was observed, as indicated by trends of modification in pro- and anti-inflammatory cytokines and chemokines in plasma, which can cause long-term alterations in nociceptive perception.

The SARS-CoV-2 infection model employed in this study was originally developed by Jiménez de Oya et al., who demonstrated the susceptibility of hACE2 transgenic mice to the Omicron variant (BA.1.17 lineage) [17]. In this experimental model, infected mice exhibited detectable viral RNA in the lungs at 4 days post-infection, which was no longer present 15 days after. Also, in this animal model, histopathological analysis revealed inflammatory lesions in the lungs, supporting its relevance for investigating post-COVID-19 pathology [17]. In the present study, the same animal model developed by Jiménez de Oya et al. [17] was used, and behavioral assessments were conducted to evaluate nociception in animals following SARS-CoV-2 infection. Notably, to our knowledge, this is the first study to assess nociceptive behavior in a murine model of SARS-CoV-2 infection, whereas previous research explored pain mechanisms by administering purified viral proteins to non-infected mice [22]. Specifically, Cui et al. demonstrated that the SARS-CoV-2 envelope protein can induce acute pain through TLR2/NF-κB signaling in non-infected mice; however, our study is the first to explore nociceptive changes in the context of real SARS-CoV-2 infection.

### 3.1. Pain Sensitivity After SARS-CoV-2 Infection

The presence of pain sensitivity in people with post-COVID-19 condition is a topic of debate. It has been suggested that there is an increased excitability of the central nervous system in a subgroup of subjects with post-COVID-19 condition [23]. Sensitization is characterized by increased nociceptive facilitation, decreased pain thresholds, and a less efficacious descending nociceptive inhibitory system—all features scarcely investigated in post-COVID-19 conditions. For instance, Goudman et al. identified that 12–14% of individuals with post-COVID-19 condition exhibit deficient conditioned pain modulation [24]. Data on hyperalgesia in individuals with post-COVID-19 condition is conflicting and is focused on mechanical hypersensitivity. Goncalves et al. found mechanical pain hypersensitivity (i.e., lower pressure pain thresholds) in survivors developing new post-COVID-19 pain symptomatology [25], whereas Baroni et al. did not find differences in pressure pain sensitivity between individuals with and without post-COVID-19 pain [26].

In our animal model, we assessed thermal pain sensitivity to investigate the potential development of post-infection pain by measuring withdrawal thresholds to a noxious thermal stimulus using the hot-plate test. Although we observed a trend towards lower nociceptive thresholds on days 14 and 28 post-infection, overall, our findings did not indicate increased pain sensitivity in female mice infected with the Omicron variant (lineage BA.1.17). Overall, the absence of detectable changes in thermal thresholds should not be interpreted as definitive evidence of normal or other thresholds. This study was designed as a preliminary approach to explore potential nociceptive alterations following Omicron variant SARS-CoV-2 infection; hence, additional tests targeting different pain modalities (e.g., von Frey for mechanical sensitivity, cold plate, or spontaneous pain measures) were beyond its scope.

Thus, current experimental data from this animal model cannot support the presence or absence of sensitivity to pressure pain after an acute SARS-CoV-2 infection, in agreement with Baroni et al. [26] but contrary to Goncalves et al. [25]. It is possible that pressure pain sensitivity is present in a subgroup of patients and not in all individuals with post-COVID-19 condition.

### 3.2. Long-Lasting Presence of Inflammatory Biomarkers

It is suggested that neuroimmune inflammation could be one of the potential mechanisms for post-COVID-19 pain, promoting nociceptive sensitization and chronic pain. Therefore, it is suggested that several of the pro-inflammatory signaling molecules, which are elevated at the acute COVID-19 phase, impact skeletal muscle and that the SARS-CoV-2 cell-to-cell inflammatory response associated with cytokine and interleukin storms provokes hyperexcitability of the peripheral and central nervous systems, leading to the development of post-COVID-19 pain symptoms [27]. A narrative review reported that patients with long COVID showed higher levels of IL-6, IL-17, CCL3, and TNF-α, based on a small number of studies [28]. The meta-analysis by Yong et al. investigated the presence of different inflammatory biomarkers in COVID-19 survivors with/without post-COVID-19 condition [29]. From those biomarkers included in our study, this meta-analysis pooled data on IL-6 (seven studies), IL-10 (three studies), IFN-γ (three studies), and TNF-α (four studies). The overall results revealed no differences in these inflammatory biomarkers between patients with and without post-COVID-19 condition [29]. After adjusting by group and sensitivity analysis, they observed higher IL-6 levels (SMD: 0.30, 95%CI: 0.12–0.49) in patients with post-COVID-19 condition when compared with those without [29]. The presence of higher IL-6 levels in patients with post-COVID-19 condition is also supported by a meta-analysis specifically focused on this biomarker [30].

Our results did not reveal a clear pattern of changes in plasma levels of inflammatory cytokines and chemokines in infected animals compared to uninfected animals that would corroborate the findings reported in the human studies mentioned. Only when analyzing the heat map was there a tendency towards an increase in CXCL5 and CXCL2 and a decrease in CCL11, CCL24, CXCL13, IL-10, and CCL22. All of this indicates an imbalance between pro-inflammatory, reparative, and regulatory signals. The increase in chemokines such as CXCL5 and CXCL12 suggests an acute response with a strong neutrophilic component and activation of angiogenesis and tissue repair mechanisms. In contrast, the decrease in chemokines associated with eosinophil recruitment (CCL11 and CCL24) and lymphoid tissue organization (CXCL13) indicates a lower tendency towards chronic processes mediated by eosinophils and B lymphocytes. Furthermore, the reduction in regulatory cytokines such as IL-10 and CCL22 reflects a loss of immunoregulatory control, decreasing the ability to resolve inflammation and favoring its persistence. These changes, although only representing trends, could not be related to the heat nociception results observed in our study; however, they could indicate an inflammatory environment in the animals that could cause long-term alterations in nociceptive perception. In fact, one of the described potential mechanisms for post-COVID-19 pain is a neuroimmune response in which low-grade inflammation persists, promoting nociceptive sensitization and chronic pain. Increased CXCL5 potentiates neuronal activation and sensitization, as it has been directly associated with neutrophil-mediated inflammatory pain and peripheral sensitization [31]. On the other hand, elevated CXCL12 and its CXCL12/CXCR4 axis promote nociceptive plasticity and the maintenance of chronic pain through a positive feedback loop in pain synapses [32,33]. Furthermore, the reduction of IL-10 and CCL22 decreases anti-inflammatory and regulatory mechanisms, favoring a state of chronic inflammation that perpetuates central and peripheral pain sensitization [34]. Taken together, this profile—with increased pro-nociceptive chemokines and decreased regulatory mediators—suggests a solid biological basis for persistent post-COVID-19 pain, characterized by hypersensitivity, altered nerve function, and possible prolonged neuroinflammation. In any case, the changes are complex, and the presence of inflammatory biomarkers in post-COVID-19 conditions deserves further study.

### 3.3. Nerve Damage After SARS-CoV-2 Infection

Some case reports have described the development of neuropathic pain conditions such as post-herpetic neuralgia, trigeminal neuralgia, or brachial plexopathy after an acute SARS-CoV-2 infection [35]. According to the International Association for the Study of Pain (IASP), neuropathic pain is diagnosed when (1) an identifiable lesion/disease of the somatosensory nervous system (i.e., central or peripheral nervous system) is identified; (2) pain is limited to a “neuroanatomically plausible” distribution of the nervous system; and (3) pain is supported by clinical examination findings as well as imaging and/or laboratory findings [36]. Several studies have investigated the prevalence of neuropathic pain-associated symptoms in individuals with post-COVID-19 condition, but more research is needed. In fact, two meta-analyses reported pooled prevalence rates of neuropathic post-COVID-19 pain symptoms ranging from 10% (95%CI: 5–15%) [37] to 34.3% (95%CI: 14.3–62%) [38]. These discrepancies can be related to the fact that most studies have not identified the presence of nerve damage in their patients, and the data are mostly self-reported.

The presence of nerve inflammation and damage would explain the development of some peripheral neuropathies observed in COVID-19 survivors. Thus, evaluating the tissue expression of IL-6, IL-1β, and IL-18 in a sensory nerve could provide information about neuroinflammation related to neuropathic pain in post-COVID-19 conditions. IL-1β, by binding to its receptor in nociceptors, induces peripheral sensitization, being responsible for mechanical hyperalgesia in models of chronic inflammation [39]. Similarly, IL-6 modulates neuronal activity and promotes changes in neuronal excitability that can perpetuate pain signals in post-viral contexts [40]. Finally, IL-18 acts as a central node in peripheral nerve neuroinflammation [41]. Measuring these cytokines would localize the neuroimmune connection between systemic inflammation and local alteration in the sensory nervous system, providing a solid justification for possible therapies targeting these mediators in the context of post-COVID-19 pain. However, our results did not reveal any expression of IL-6, IL-1β, and IL-18 in the saphenous nerve in either infected or non-infected animals. The lack of correlation between plasma inflammatory biomarker trends and the absence of local inflammation in the peripheral nerve may account for the unchanged thermal sensitivity observed. We also do not know whether other underlying mechanisms, such as muscle damage or small fiber neuropathy, could be involved in the development of post-COVID-19 pain [42]. Finally, it should be noted that, given that there are no studies in the current literature on infected animals that analyze this aspect, further studies are needed to confirm or refute this fact.

### 3.4. Strengths and Limitations

To our knowledge, this is the first study to evaluate nociceptive responses in SARS-CoV-2-infected animals, as the limited evidence available to date is based on uninfected animal models, which may not accurately simulate the nociceptive alterations resulting from infection. Furthermore, most animal models of COVID-19 have been extensively used to study viral susceptibility, pathogenicity, and transmission, but not nociception. It should be noted that animals in this model were infected with the Omicron variant of concern. Although Omicron is the current predominant SARS-CoV-2 variant, it is important to note that each variant of concern shows different tropism, replication capacity, and inflammatory profile and can lead to different post-COVID-19 symptomatology [43]. In fact, current data suggest that the Omicron variant leads to a lower prevalence of post-COVID-19 symptomatology than previous variants of concern, e.g., Alpha or Delta, as well as the historical strain [44]. However, there is still a lack of systematic comparative studies between variants of concern, such as Alpha, Delta, or Omicron, in animal models.

Several limitations of this animal study should be considered. In humans, post-COVID-19 pain is predominantly associated with deep-somatic tissues; therefore, the use of an acute, cutaneous nociceptive stimulus in animals may not fully capture the complexity of this condition (e.g., mechanical allodynia). A priori power calculations were not performed for the current study due to its explorative character, including a broad range of proteins. We acknowledge the relatively small sample size due to the loss of five animals during the first week post-infection, which may have reduced the statistical power of some analyses. It is also possible that the animals euthanized early due to severe clinical signs could have exhibited more pronounced biomarker changes, which we were unable to assess.

Finally, in relation to the biomarkers analyzed, studying the expression of IL-6, IL-1β, and IL-18 in the saphenous nerve allowed us to verify whether the systemic inflammation observed also affected the nervous tissue. This is important because local neuroinflammation is what really contributes to sensitization and chronic post-COVID-19 pain. However, the study of biomarkers could have been extended to other relevant cytokines, such as TNF-α, IL-8, or even chemokines such as CXCL12 and CXCL5, which could be measured in the nerve, as they are involved in the activation of nociceptors and the migration of immune cells to sensory ganglia. In addition, other neuronal tissues (dorsal root ganglia and spinal cord) could also be analyzed to complete the study.

## 4. Methods

### 4.1. Ethics Statement

The study was conducted in accordance with the Ethical Committee of Animal Experimentation of INIA-CSIC and the Division of Animal Protection of the Comunidad de Madrid (PROEX 115.5-21), and it was approved by the Local Research Ethics Committee of the Universidad Rey Juan Carlos (Madrid, Spain; code: ENM159/202311202122021, approved on 22 January 2022). It followed the guidelines for the Care and Use of Laboratory Animals of the European Community (European Directive 2010/63/EU).

### 4.2. Model of SARS-CoV-2 Infection in Female Mice

The animal model of SARS-CoV-2 infection and experimental procedures were developed in the biosafety level 3 facilities at Centro de Investigación en Sanidad Animal, Instituto Nacional de Investigación y Tecnología Agraria y Alimentaria (INIA-CSIC), Madrid (Spain). C57BL/6 female mice were used for transgene production; transgenic mice expressing the human angiotensin-converting enzyme 2 (ACE2) receptor under the human cytokeratin 18 (K18) gene promoter’s control (hACE2) were generated [45]. Subsequently, mice were anesthetized with 5-2% of isoflurane (+0.5–1 L/min) and intranasally (i.n.) inoculated with 50 μL of the Omicron variant of SARS-CoV-2 (lineage BA.1.17, 104 TCID50/mouse) [17].

### 4.3. Experimental Design

A total of 23 hACE2 female mice (age: 6 weeks old, weighing 20–25 g) were used in the study. Those mice infected with SARS-CoV-2 (n = 12) were considered the infected group, whereas a sample of non-infected mice (n = 11) was considered the control group. All mice received food and water ad libitum and were monitored daily for clinical signs and body weight. Animals exhibiting advanced clinical signs of SARS-CoV-2 infection and significant health deterioration (weight loss exceeding 20% of initial body weight or severe symptoms such as lethargy, inactivity, hunched posture, or markedly abnormal respiration) were humanely euthanized. All animals that survived until the end of the experimental period (28 days after infection) were used in the nociception study and were subsequently anesthetized and sacrificed to carry out the biomarker study. The 28-day endpoint is approximately equivalent to three human years [46], aligning with the timespan included in the definition of post-COVID-19 condition [47].

**Figure 5:**
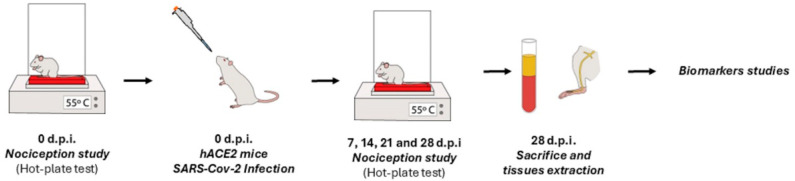
Experimental protocol. Female hACE2 mice were infected intranasally with the SARS-CoV-2 Omicron variant. Heat nociceptive thresholds (55 °C) were assessed using the hot-plate test before infection at 7-, 14-, 21-, and 28-days post-infection. After completing the nociception assessments, animals were sacrificed on day 28 for tissue collection and subsequent biomarker analysis. All these procedures were performed on all animals that survived up to 28 days after infection. An identical protocol was applied to non-infected hACE2 mice, which served as the control group.

Nociception to heat stimulus was assessed on day 0 (pre-infection) and 7-, 14-, 21-, and 28-days 5ost-infection to evaluate possible alterations in nociceptive thresholds after infection. Finally, all animals were sacrificed, blood samples were collected, and paw/saphenous nerve tissue was dissected for the biomarker analyses (Figure 5).

### 4.4. Evaluation of Nociceptive Threshold to Heat Stimulus: Hot-Plate Test

Nociceptive threshold to heat was measured in the hot-plate test before (0) and 7, 14, 21, and 28 days after the infection as follows: mice were placed on the hot-plate (hot/cold plate, Ugo Basile, Gemonio, Italy), programmed to 55 °C, in a Plexiglas cylinder. The latency (s) to the first nociceptive response (licking of forepaws or jumping) was taken as an index of nociceptive threshold. A cut-off time of 30 s was established to avoid damage to the paw [48]. The nociceptive threshold is expressed as a percentage, considering that 100% is the latency on day 0 (before infection).

### 4.5. Plasma Inflammatory Marker Multiplex Analysis

To analyze the possible existence of chronic systemic inflammation in animals 28 days post-infection, plasma protein levels of cytokines and chemokines were assessed using the Bio-Plex MAGPIX, based on the Luminex assay. A ProTM Mouse Cytokine 31-Plex Assay kit (Bio-Rad, Hercules, CA, USA, Cat#12009159) was employed following the manufacturer’s instructions. This assay included the detection of cytokines (granulocyte-macrophage colony-stimulating factor/GM-CSF), interferon (IFN)-γ, interleukins (IL-1β, IL-2, IL-4, IL-6, IL-10, IL-16), tumor necrosis factor-α (TNF-α), chemokines (c-c motif family: CCL1, CCL2, CCL3, CCL4, CCL5/RANTES, CCL7, CCL11/Eotaxin, CCL12, CCL17, CCL19, CCL20, CCL22, CCL24/Eotaxin2, CCL27/CTACK, and CXCL family (CXCL1, CXCL5/ENA-78, CXCL10, CXCL11, CXCL12, CXCL13/BCA-1, CXCL16, and CX3CL1/fractalkine)). The determination of cytokines/chemokines in each sample was performed in duplicate. Following, levels of proteins were calculated via Bio-Plex ProTM software (Bio-Rad Laboratories, Inc., Hercules, CA, USA) according to standard curves for each protein, generated using a kit-supplied reference cytokine/chemokine sample [49].

The trend fold change for each sample was calculated as the ratio between the concentration of each sample of the infected group and the mean concentration of the control non-infected group. The fold changes were transformed to a log2 scale to accommodate the dynamic range in the concentration values [50].

### 4.6. Immunohistochemical Analysis in Saphenous Nerve

To evaluate the possible link between systemic inflammation and local neuroimmune alteration, potential inflammatory biomarkers (IL-1β, IL-6, and IL-18), related to nociception [39,40,41] in the context of SARS-CoV-2 infection, were analyzed. For that, the chosen nerve tissue was the saphenous nerve, as it is a purely sensory branch of the femoral nerve, devoid of motor fibers, allowing for a specific evaluation of nociceptive mechanisms without motor confounding factors [51,52].

Immediately after animals were euthanized, the hind limb was extracted and fixed in 10% formalin. To isolate the saphenous nerve, the limb was stabilized on a support platform, and the nerve was carefully dissected along its entire length, in block with the surrounding muscle tissue and blood vessels, using a scalpel. Nerve samples were preserved in 10% formalin and placed in histological cassettes for further processing.

Dehydration and paraffin embedding of the saphenous nerve samples were performed using an automatic Citadel 2000 Tissue Processor (Thermo Fisher Scientific, Oxford, UK), following a standard protocol involving sequential immersion in alcohol solutions of increasing concentration (70%, 90%, and absolute), toluene, and paraffin, with each step repeated three times. Samples remained in liquid paraffin for 24 h. Subsequently, the cassettes were transferred to an oven at 60 °C for an additional 24 h. After this period, samples were embedded in paraffin using a paraffin dispenser, forming solid blocks with the tissue included. These blocks were sectioned using a Micron HM360 microtome (Leica Microsystems, Wetzlar, Germany), and the sections were mounted on slides treated with poly-L-lysine. Finally, the slides were incubated in an oven for 24 h to ensure proper tissue adhesion.

A representative paraffin block from each case was chosen and subjected to immunohistochemistry for IL-6, IL-1β, and IL-18 cytokines. Histological sections were deparaffinized and rehydrated before endogenous peroxidase activity was blocked with 0.3% H_2_O_2_ in methanol. The slides were rinsed with PBS-Tween, soaked in serum for 20 min, and incubated with primary antibodies in a moist chamber for 24 h in the refrigerator. The sections were subsequently incubated with anti-mouse for 30 min at room temperature, rinsed with PBS, and fully covered by DAB + H_2_O for 10 min. Afterward, the slides were immersed in hematoxylin for 1 min for counterstaining, and subsequently placed in different containers for 1 min each to achieve dehydration. The immunostaining reaction product was developed using diaminobenzidine. The primary antibodies used were anti-IL-6 (Abcam, [EPR23819-103, 0.575–0.596 mg/mL], 1:100), anti-IL-1β (Abcam, [RM1009] 1/100), and IL-18 (Invitrogen, Carlsbad, CA, USA, Catalog # PA5-79482; 1:500).

All histological slides were studied under a Zeiss Axiophot 2 microscope (Carl Zeiss AG, Jena, Germany) and photographed with an Axiocam HRc camera (Carl Zeiss AG, Jena, Germany). All the histological slides were evaluated by the same researcher without knowledge of the groupings.

### 4.7. Statistical Analysis

The statistical analysis was performed using GraphPad Prism software version 8.0 (San Diego, CA, USA). Kaplan–Meier survival analysis was performed for survival curves. All data were checked for normality by the Shapiro–Wilk test and expressed as mean ± standard error of the mean (SEM). To compare between-groups differences, unpaired Student’s *t*-test or two-way repeated measures ANOVA, followed by Sidak’s multiple comparison test, were used. The histological analysis assessed the differences between groups using the corrected chi-squared test. *p* < 0.05 values were considered statistically significant.

## 5. Conclusions

In this post-COVID-19 animal model, female transgenic mice infected with the SARS-CoV-2 Omicron variant did not develop thermal sensitization, nor did they exhibit significant alterations in inflammatory or nerve damage biomarkers at 28 days post-infection. However, the observed existence of a slight inflammatory environment in animals, as indicated by trends of modification in pro-inflammatory and anti-inflammatory cytokines and chemokines in plasma, may cause long-term alterations in nociceptive perception. The absence of detectable changes in this animal model underscores the need for further research to clarify the discrepancies observed in human patients and to explore alternative pathways potentially involved in post-COVID-19 pain syndromes.

## Figures and Tables

**Table 1:** Overview of plasma cytokines and chemokines levels using the Bio-Plex assay in infected (n = 7) and non-infected control (n = 11) groups. Data are presented as mean ± SEM.

Analyte	Infected Group (n = 7)	Non-Infected Group (n = 11)	*p*-Value
GM-CSF	28.16 ± 3.45	25.07 ± 3.49	0.5591
IFN-γ	327.0 ± 52.06	285.10 ± 26.59	0.4405
IL-1β	213.30 ± 30.86	198.80 ± 14.39	0.6461
IL-2	15.39 ± 2.69	14.74 ± 1.52	0.8251
IL-4	146.6 ± 25.85	133.0 ± 12.73	0.6067
IL-6	120.7 ± 20.96	102.50 ± 13.71	0.4572
IL-10	3369.0 ± 581.40	3493.0 ± 270.0	0.8293
IL-16	600.40 ± 93.89	563.90 ± 59.34	0.7337
TNF-α	615.40 ± 92.07	572.30 ± 57.43	0.6809
CCL1	56.86 ± 8.22	49.48 ± 5.03	0.4270
CCL2	1033.0 ± 126.80	950.10 ± 77.20	0.5622
CCL3	92.50 ± 12.03	84.82 ± 7.04	0.5623
CCL4	267.4 ± 20.32	251.0 ± 26.25	0.6634
CCL5/RANTES	76.42 ± 10.20	73.08 ± 7.79	0.7963
CCL7	186.60 ± 24.55	168.40 ± 13.97	0.4964
CCL11/Eotaxin	140.90 ± 25.55	189.0 ± 27.85	0.2533
CCL12	41.13 ± 3.63	35.64 ± 2.63	0.2283
CCL17	829.40 ± 162.30	721.40 ± 79.97	0.5159
CCL19	3440.0 ± 416.30	3064.0 ± 307.30	0.4695
CCL20	74.19 ± 7.87	69.46 ± 4.80	0.5931
CCL22	451.50 ± 49.53	495.30 ± 46.34	0.5427
CCL24/Eotaxin2	5065.0 ± 433.20	6218.0 ± 581.90	0.1748
CCL27/CTACK	2864.0 ± 331.60	2543.0 ± 196.40	0.3851
CXCL1	943.10 ± 86.90	910.0 ± 61.07	0.7520
CXCL5/ENA-78	22,633.0 ± 1639.0	15,798.0 ± 2041.0	0.0726
CXCL10	3490.0 ± 333.6	3274.0 ± 261.0	0.6165
CXCL11	3973.0 ± 405.10	3760.0 ± 452.20	0.7504
CXCL12	1363.0 ± 129.2	1321.0 ± 86.33	0.7858
CXCL13/BCA-1	1383.0 ± 154.0	1602.0 ± 161.40	0.2944
CXCL16	466.0 ± 34.53	481.8 ± 48.18	0.8163
CX3CL1/Fractalkine	222.50 ± 23.36	211.80 ± 14.10	0.6837

## Data Availability

All data derived from this study are presented in the text.
